# Hesperidin reduces genomic instability in glioblastoma cells and is associated with reduced HNRNPA2B1/m^6^A levels

**DOI:** 10.3389/fphar.2026.1876112

**Published:** 2026-09-08

**Authors:** Viktoriia Sazonova, Pavel Zun, Ilia Tonkji, Sergey Onikienko, Tatyana Erofeeva, Vadim Basharin, Nadezhda Zhigalova, Alexandra Ilyushkina, Ekaterina Skorb, Alexey Ruzov, Sergey Shityakov, Viacheslav Kravtsov

**Affiliations:** 1 Infochemistry Scientific Center, ITMO University, Saint Petersburg, Russia; 2 Personalized Biomedical Technologies Sector of the Saint Petersburg Research Center of the Russian Academy of Sciences, Saint Petersburg, Russia; 3 Department Toxicology of Medical Academy, Saint Petersburg, Russia; 4 Institute of Bioengineering, Research Center of Biotechnology, Russian Academy of Sciences, Moscow, Russia; 5 Department of Biology and General Genetics, Sechenov First Moscow State Medical University (Sechenov University), Moscow, Russia

**Keywords:** epitranscriptomics, genomic instability, glioblastoma, hesperidin, HNRNPA2B1, ionizing radiation, M6A RNA methylation, micronuclei

## Abstract

Genomic instability contributes to tumor progression and limits the effectiveness of anticancer therapy. In this study, we investigated whether the citrus flavonoid hesperidin influences cytogenetic damage and epitranscriptomic markers in tumor cells exposed to ionizing radiation or maintained under long-term culture conditions. In human glioblastoma cell lines U87MG and LN229, hesperidin reduced radiation-induced micronucleus formation and decreased the spontaneous micronucleus frequency during prolonged passaging with repeated supplementation. Immunocytochemical analysis demonstrated that long-term hesperidin treatment was accompanied by reduced HNRNPA2B1 protein expression and lower global m^6^A RNA levels. In a primary culture of collecting duct renal carcinoma cells, hesperidin altered cell morphology and growth dynamics during long-term cultivation. Together, these findings indicate that hesperidin reduces markers of genomic instability in tumor cell models and is accompanied by changes in HNRNPA2B1 expression and global m^6^A RNA methylation. The mechanistic relationship between these observations requires further investigation.

## Graphical Abstract

**Figure:**
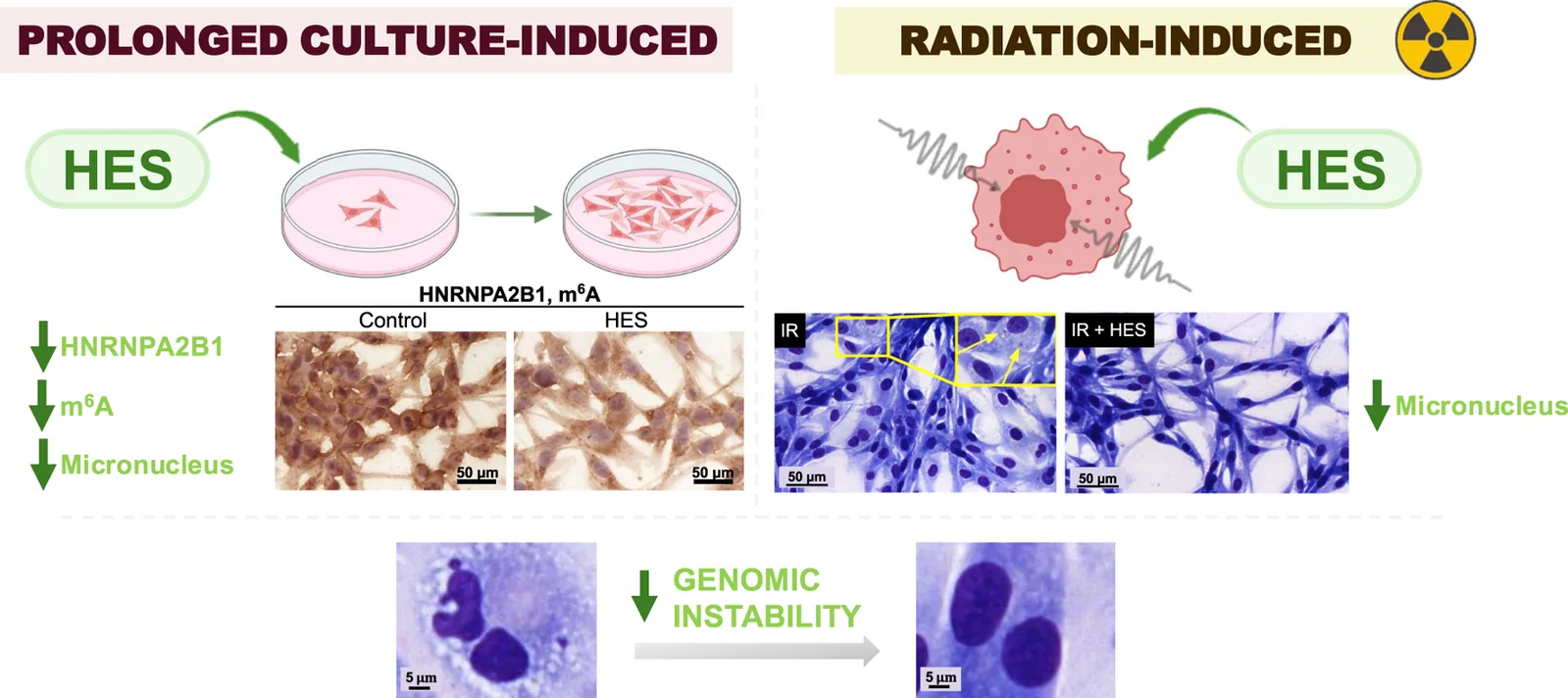

## 1 Introduction

Modern cancer biology considers genomic instability to be one of the key mechanisms of tumor progression. It provides tumor cells with genetic diversity and contributes to the formation of distinctive features of cancer, including evasion of apoptosis, enhanced proliferation, angiogenesis, and development of chemoresistance (Hanahan and Weinberg, 2011; Negrini et al., 2010). The most pronounced genomic instability is observed in glioblastoma, an aggressive brain tumor characterized by chromosomal rearrangements, aneuploidy, and mutations in TP53, EGFR, and PTEN (Mazzoleni et al., 2024; Crespo et al., 2015). One of the central sources of such disorders are mitotic errors and the formation of micronuclei, leading to chromothripsis and other forms of chromoanagenesis (Liu et al., 2014; Holland and Cleveland, 2012; Pellestor, 2019; Crasta et al., 2012).

Understanding the molecular mechanisms underlying genomic instability has led to the identification of new therapeutic targets, among which RNA-binding proteins of the HNRNP family are of particular interest. The HNRNPA2B1 protein, one of the main readers of post-transcriptional m^6^A modification, regulates the splicing, export, and stability of oncogenic transcripts (Li et al., 2022; Jin et al., 2024; Chen et al., 2019). Its overexpression has been demonstrated in various tumors, including glioma, lung cancer, liver cancer, breast cancer, pancreatic cancer, and multiple myeloma (Hu et al., 2024; Jia et al., 2022; Wang et al., 2018; Fielding et al., n.d.), and a decrease in its activity increases the sensitivity of tumor cells to chemotherapy. This makes HNRNPA2B1 a promising target for therapy aimed at normalizing genomic stability.

Alongside the development of experimental methods, the role of computational technologies–in particular, molecular docking–is growing. This tool allows prediction of the interaction of potential drug molecules with proteins and effective repurposing of existing drugs in new therapeutic scenarios. The high accuracy of modern docking algorithms makes the method valuable for identifying inhibitors, including natural compounds suitable for radiomodulation (Li et al., 2021; Shityakov and Foerster, 2014; Kovalenko et al., 2023; Tamaian et al., 2023).

In the search for safe radioprotectors and radiosensitizers, natural substances are attracting increasing attention, especially flavonoids–compounds with a broad spectrum of antioxidant, anti-inflammatory, and antitumor activity (Comalada et al., 2006; Persano et al., 2022; Kopustinskiene et al., 2020). Among them, hesperidin is one of the most studied due to its ability to suppress pro-inflammatory signaling pathways, neutralize free radicals, and reduce the damaging effects of ionizing radiation. Preclinical studies show that hesperidin protects tissues from oxidative stress, reduces the frequency of radiation-induced micronuclei, and decreases the biochemical and histological signs of radiation damage (Mun et al., 2018; Kalpana et al., 2011; Pradeep et al., 2012; Hosseinimehr et al., 2009).

Despite the growing body of data, whether hesperidin can directly affect the level of genomic instability of malignant cells and whether its effects correlate with changes in the HNRNPA2B1/m^6^A regulatory axis has hardly been studied. In our recent *in silico* study, we performed structure-based molecular docking to evaluate the potential binding of hesperidin to HNRNPA2B1 and identified the most probable interaction sites. The modeling predicted a favorable binding mode of hesperidin within the RRM domain, stabilized by multiple hydrogen bonds and hydrophobic contacts with key amino acid residues, and demonstrated strong binding affinity. These computational results suggested that HNRNPA2B1 may represent a potential molecular target of hesperidin (Lubinets et al., 2026). Based on these observations, the present study aimed to investigate whether hesperidin treatment is associated with changes in genomic instability, HNRNPA2B1 protein expression, and global m^6^A RNA methylation in tumor cell models under radiation exposure and long-term cultivation conditions.

## 2 Materials and methods

### 2.1 Hesperidin source and quality control

Hesperidin was obtained from a commercially available dietary supplement (NaturalSupp, Russia). Each capsule was labeled by the manufacturer to contain 400 mg of hesperidin. For experimental use, the capsule contents were dissolved in sterile distilled water to a nominal concentration of 8 mg/mL. Additional analytical characterization of the commercial hesperidin preparation by LC-MS/MS, including confirmation of compound identity, is described in Section 2.7. Based on a 70 μL addition of the 8 mg/mL stock solution to 5 mL of culture medium, the final nominal hesperidin concentration was approximately 112 μg/mL (∼184 μM).

### 2.2 Culturing and treatment of glioblastoma cell lines

The experimental part of the study was conducted using two widely applied human glioblastoma cell lines, U87MG and LN229, obtained from the American Type Culture Collection (ATCC, University Boulevard, 10,801, Manassas, VA 20110-2209, USA). Cells were cultured in Dulbecco’s Modified Eagle’s Medium (DMEM, BioloT, Russia) supplemented with 10% fetal bovine serum (FBS, BioloT, Russia) and 1% (v/v) penicillin-streptomycin (10,000 U/mL stock, BioloT, Russia), yielding a final concentration of 100 U/mL penicillin and 100 μg/mL streptomycin. Cultivation was performed under standard conditions at 37 °C in a humidified atmosphere containing 5% CO_2_. Cell density was monitored visually using phase-contrast microscopy with an inverted microscope, and images were documented for subsequent analysis.

For modeling radiation exposure and assessing the radiosensitivity of tumor cells, X-ray irradiation was carried out using a mobile X-ray system PRDU (CJSC “ELTECH-Med”, Russia). Cells were exposed to doses of 0 Gy, 1 Gy (100 kV, 10 mA, 38 s), 2 Gy (100 kV, 10 mA, 76 s), 4 Gy (100 kV, 10 mA, 153 s), and 8 Gy (100 kV, 10 mA, 306 s), in accordance with commonly accepted experimental protocols (Ye et al., 2014). Prior to irradiation, cells in the experimental group were incubated for 3 h with the hesperidin working solution. Both control and experimental cells were subjected to identical culture and irradiation conditions. After irradiation, cells were transferred onto glass slides for fixation and staining. Samples were air-dried at room temperature, fixed with 70% ethanol to preserve nuclear and cytoplasmic structures, and subsequently stained using the Romanowsky method with azure-2/eosin solution (Biovitrum, Russia) diluted 1:10 with distilled water. The stain was applied evenly over the smear for 5 min, followed by rinsing in distilled water and air-drying. Microscopic analysis was performed using an inverted microscope LEICA DMi 8 (Leica Microsystems GmbH) equipped with Leica Application Suite software. Imaging was carried out at ×40 (objective 0.65 PH2) and ×100 (objective 1.25 OIL PH3, immersion oil) magnifications, enabling high-resolution visualization of morphological features. Analysis included detection of genomic instability markers such as micronuclei formation, multinucleated cells, nuclear shape abnormalities, apoptosis, and mitotic defects. Observed changes were documented by digital microphotography for further quantitative analysis. Additionally, all prepared samples were digitized using a slide scanner provided by collaborating institutions. Acquired images were processed using the Cellpose + algorithm (Huaman et al., 2025), ensuring accurate recognition, segmentation, and automated quantification of genomic instability markers. After segmenting the images with Cellpose+ and manually checking the obtained segmentations to ensure correctness, nuclei smaller than 30 μm^2^ were classified as micronuclei. The percentage of micronuclei among the total segmented number of nuclei was used as a measure of genome instability.

### 2.3 Hesperidin treatment and post-irradiation analysis

Three hours after incubation with hesperidin, all culture flasks–both experimental and control–were subjected to X-ray irradiation as described in Section 2.2. Following irradiation, cells were returned to standard incubation conditions (37 °C, 5% CO_2_) for 72 h.

At the end of incubation, cells from each group were detached using trypsin-versene solution (BioloT, sterile, 1:1) for 5 min. Detached cells were collected into tubes, centrifuged at 1550 *g* for 4 min, and the pellet was resuspended in serum-free DMEM to remove residual FBS that could interfere with subsequent staining procedures. Experiments with hesperidin treatment followed by irradiation of U87MG and LN229 cells were performed in triplicate to ensure reproducibility.

### 2.4 Dose–response curves for micronuclei frequency and HNRNPA2B1 protein expression

Glioblastoma cell cultures were irradiated at doses of 0, 1, 2, 4, and 8 Gy. After irradiation, cells were incubated for 72 h under standard conditions (37 °C, 5% CO_2_). At the end of incubation, cells were collected, treated with trypsin-versene, and prepared for staining to determine the frequency of micronucleus formation.

For HNRNPA2B1 protein expression analysis, an identical experiment was performed in 12-well plates. After 72 h incubation, cells were fixed with 4% paraformaldehyde (PFA) and stained according to a standard immunofluorescence protocol (Abakir et al., 2020). Optical sections (500 nm wavelength) were acquired using a Zeiss LSM 700 AxioObserver confocal microscope with a Plan-Apochromat 63×/1.40 Oil DIC M27 objective and analyzed with ImageJ and Adobe Photoshop software. Immunofluorescence was selected for this experiment to enable high-resolution analysis of HNRNPA2B1 subcellular localization and signal intensity following radiation exposure.

### 2.5 Long-term effect of hesperidin on genomic stability of glioblastoma cells: micronucleus assay

To assess the long-term effect of hesperidin on genomic stability, extended experiments were carried out using the U87MG cell line. Hesperidin working solution was added to culture flasks at a nominal concentration of 8 mg/mL in 70 µL volume. Treatment was repeated at every passage, with the total cultivation period comprising 20 passages. Control cells were passaged in parallel under identical conditions without hesperidin supplementation.

After every fifth passage (passages 5, 10, 15, and 20), a subset of cells from both control and hesperidin-treated groups was harvested simultaneously and analyzed by micronucleus assay as described previously. Samples were fixed, stained, and evaluated for micronuclei frequency as a marker of genomic instability. This approach allowed evaluation of cumulative hesperidin effects on genomic stability in glioblastoma cell populations while controlling for any passage-dependent effects.

### 2.6 Immunocytochemical staining for HNRNPA2B1 protein and m^6^A-modified RNA in glioblastoma cells

Immunocytochemical staining was performed to examine HNRNPA2B1 protein expression and global RNA m^6^A methylation levels in U87MG cells following long-term hesperidin treatment. Cells were cultured for 3 days on coverslips in the presence or absence of hesperidin. Following incubation, cells were fixed and treated with 3% hydrogen peroxide (H_2_O_2_) for 10 min in a humid chamber to block endogenous peroxidase activity. Samples were then rinsed with phosphate-buffered saline (PBS, pH 7.4).

Next, cells were incubated with primary antibodies against HNRNPA2B1 (rabbit anti-HNRNPA2B1, Abcam) and m^6^A-modified RNA for 30 min at room temperature. After PBS washing, signal detection was performed using a polymer-based histochemical system (Histofine Simple Stain MAX PO (MULTI), Nichirei Biosciences), suitable for mouse and rabbit antibodies (Ramos-Vara, 2005). A chromogenic DAB detection method was chosen for this experiment because it provides excellent stability for semi-quantitative population-level scoring of protein expression. Visualization was carried out using 3,3′-diaminobenzidine (DAB) substrate, diluted at one drop per 1 mL buffer. The staining reaction was monitored visually for 3–5 min until brown coloration appeared. Slides were then rinsed in distilled water and counterstained with Carazzi’s hematoxylin, enabling visualization of nuclei (blue) and localization of target deposits (brown) in the cytoplasm and/or nucleus. Immunocytochemistry was evaluated semi-quantitatively using the H-score method (Reiner et al., 1986), widely applied in clinical pathology. Staining intensity was assessed on a four-point scale: 0 – negative, 1 – weak, 2 – moderate, 3 – strong. The H-score was calculated using the formula:

$$HScore=\sum \left(i\times P\left(i\right)\right)$$

where i = staining intensity (0–3) and P(i) = percentage of cells at that intensity. The maximum possible value is 300 (if 100% of cells show intensity 3). One hundred cells from different fields were analyzed per sample. H-score values were interpreted as follows: 0–10 as negative, 10–100 as weakly positive, 100–300 as positive. For the assessment of global m^6^A levels, the H-score method was adapted to evaluate the overall intensity and distribution of the DAB signal across the cell population. This method enabled objective comparison of HNRNPA2B1 expression and global m^6^A levels between samples. Note that the quantification methods used for the radiation dose-response experiment (immunofluorescence, Section 2.4) and the long-term hesperidin experiment (chromogenic DAB with H-score, this section) are different; results from these two methods are therefore complementary but should not be directly compared quantitatively.

### 2.7 Verification of hesperidin identity by LC-MS/MS

To confirm the identity of hesperidin in the commercial dietary supplement used in this study, the preparation was analyzed by liquid chromatography coupled with tandem mass spectrometry (LC-MS/MS). The capsule contents were dissolved in methanol to obtain a stock solution at a concentration of 20 μg mL^-1^ and subsequently diluted twofold with water containing 0.1% formic acid to a final concentration of 10 μg mL^-1^ in a water–methanol mixture (1:1, v/v). Prior to analysis, the solution was filtered through a 0.22 μm membrane filter.

Chromatographic separation was performed on a Phenomenex Luna C18(2) column (150 × 2.0 mm, 5 μm, 100 Å; Phenomenex, Torrance, CA, USA) maintained at 35 °C. The mobile phase consisted of (A) water containing 0.1% formic acid and (B) acetonitrile, delivered at a flow rate of 0.3 mL min^-1^ using the following gradient: 5% B (0–2 min), linear increase to 95% B (2–15 min), 95% B (15–18 min), return to 5% B (18.1 min), followed by re-equilibration at 5% B until 23 min.

Mass spectrometric analysis was carried out using a Shimadzu LCMS-8030 triple quadrupole mass spectrometer equipped with an electrospray ionization (ESI) source operating in polarity-switching mode. Full-scan MS spectra were acquired over an m/z range of 70–1500 in both positive and negative ionization modes. Targeted analysis of hesperidin was performed in negative ion mode using multiple reaction monitoring (MRM). The deprotonated molecular ion [M−H]-(m/z 609.1) was selected as the precursor ion. Collision-induced dissociation generated three characteristic product ions (m/z 301.1, 164.1, and 151.1). The transition m/z 609.1–301.1 was used as the quantifier transition, whereas the transitions m/z 609.1–164.1 and m/z 609.1–151.1 served as qualifier transitions for confirmation of compound identity.

Raw LC-MS/MS data were converted to the mzML format for further processing. Total ion chromatograms (TIC) and extracted MRM chromatograms were analyzed, and peak areas were determined following baseline correction by trapezoidal integration. The identity of hesperidin was confirmed based on the combination of chromatographic retention time, precursor ion mass, characteristic fragmentation pattern, and qualifier-to-quantifier ion ratios, which were consistent with published LC-MS/MS data for hesperidin.

## 3 Results and discussion

### 3.1 Dose–response calibration curve for micronuclei frequency in glioblastoma cell culture

In cytological preparations of untreated and irradiated U87MG glioblastoma cell cultures, stained with azure-2 and eosin, cells with micronuclei were identified, differing in their frequency of occurrence. All detected micronuclei were localized in the cytoplasm of morphologically intact cells, which were not damaged during preparation, fixation, and staining.

**FIGURE 1:**
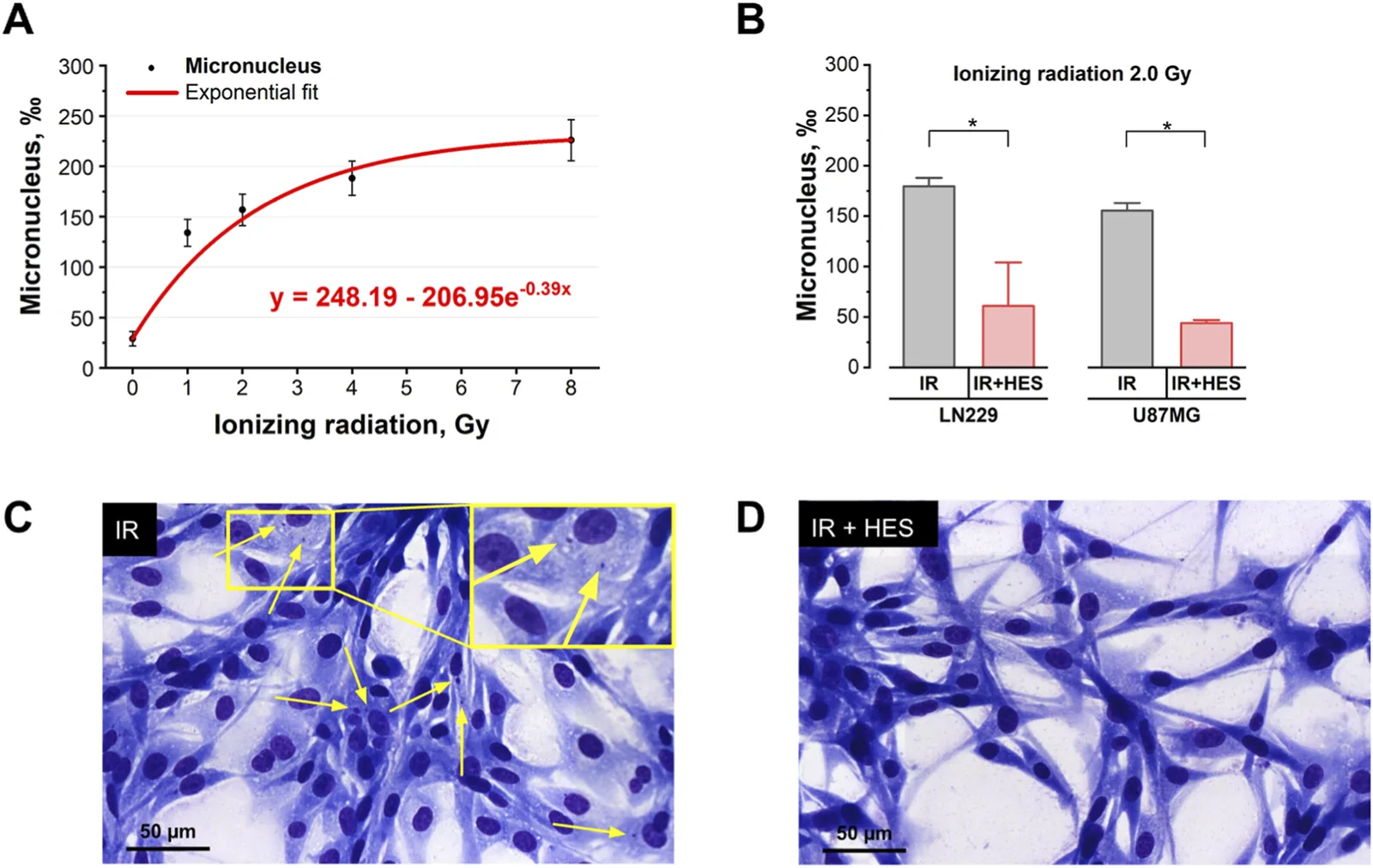
Hesperidin reduces radiation-induced micronucleus formation in glioblastoma cells. **(A)** Dose-response curve illustrating the frequency of micronuclei in U87MG cells in response to increasing doses of ionizing radiation (0–8 Gy). Data points represent mean ± SD of three independent experiments and were fitted using an exponential function. **(B)** Quantitative analysis of micronucleated cell frequency (‰) in LN229 and U87MG cell lines 72 h after irradiation (2 Gy) with or without hesperidin (HES) pretreatment. Hesperidin was added to the culture medium 3 h prior to irradiation. Data are presented as mean ± SD of three independent experiments (n = 3). Statistical significance was determined using Student’s t-test (*p < 0.05). **(C,D)** Representative microphotographs of U87MG cells (azure-2/eosin staining) from the control irradiated group **(C)** and the hesperidin-pretreated irradiated group **(D)**. Arrows indicate cells with micronuclei. Images were acquired at ×40 magnification. Scale bar: 50 μm.

The calibration curve (Figure 1A) clearly reflects the radiosensitivity of the micronucleus frequency parameter. The obtained data confirm the presence of a classical cytogenetic response to external irradiation, manifested as chromosomal aberrations, making this parameter a reliable biomarker of radiation exposure.

Based on the experimental results, a substantial increase in micronuclei frequency was observed across the entire tested dose range. A dose of 2.0 Gy was chosen as optimal for further experiments on the radiomodulating effects of hesperidin, as it induced a robust and reproducible increase in micronuclei formation without causing excessive cell death, allowing clear detection of any modifying effect.

### 3.2 Effect of hesperidin on micronuclei frequency in irradiated glioblastoma cultures

The first experiment evaluating the effect of hesperidin on micronuclei frequency in U87MG and LN229 glioblastoma cultures is presented in Figure 1B. Hesperidin was added to the culture medium 3 hours prior to X-ray irradiation at a dose of 2.0 Gy. The results demonstrated a pronounced radioprotective effect: in both cell lines, approximately a threefold reduction in micronuclei frequency was observed compared to the corresponding irradiated control groups without hesperidin. This effect was reproduced in two subsequent independent experiments, confirming the reproducibility of the results.

Thus, experimental evidence was obtained that hesperidin exhibits high efficiency in stabilizing the genome of glioblastoma cells subjected to X-ray-induced DNA damage. The next stage of the study focused on whether hesperidin could reduce spontaneous genomic instability in glioblastoma cells in the absence of external irradiation.

### 3.3 Genome stabilization by hesperidin during long-term exposure in U87MG cultures

A series of experiments demonstrated that long-term cultivation of U87MG glioblastoma cells in the presence of hesperidin was associated with a progressive reduction in spontaneous micronucleus formation, a widely used marker of genomic instability (Shityakov et al., 2023).

**FIGURE 2:**
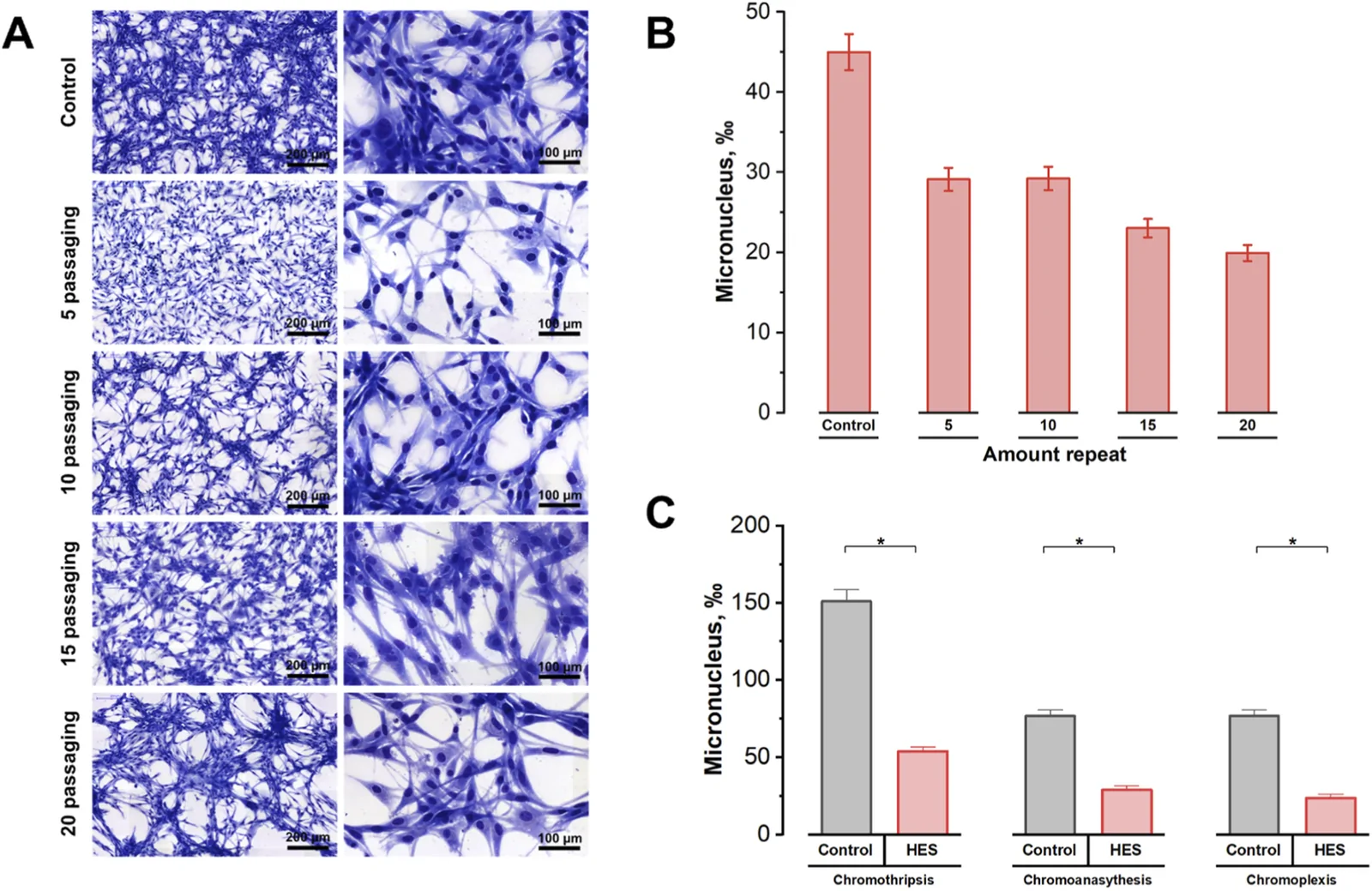
Hesperidin reduces the spontaneous frequency of micronucleated cells in U87MG glioblastoma culture during long-term *in vitro* cultivation. **(A)** Representative microphotographs of U87MG cells (azure-2/eosin staining) illustrating cell morphology and the presence of micronuclei (indicated by arrows) under control conditions and during cultivation in medium supplemented with hesperidin over 5, 10, 15, and 20 passages. All control and treated cells were derived from parallel cultures passaged simultaneously. **(B)** Passage-dependent dynamics of micronucleated cell frequency (%) in U87MG culture during long-term cultivation in control conditions and in the presence of hesperidin (HES). The analysis includes control and hesperidin-treated cells after 5, 10, 15, and 20 passages. Data are presented as mean ± SD from three independent experiments and represent simultaneous harvests from parallel cultures. **(C)** Frequency of micronucleated cells (%) and assessment of complex genomic instability events (chromothripsis, chromoanasynthesis, chromoplexis), detected by the micronucleus test, in U87MG culture after 15 passages under the indicated conditions. Data for micronucleated cells are presented as mean ± SD of three independent experiments. *p < 0.05 for genomic instability events compared to control (Student’s t-test).

Micronuclei frequency was used as the principal marker of genomic instability and was analyzed in three independent experimental series. Cultures were maintained with hesperidin and evaluated simultaneously with parallel control cultures at passages 5, 10, 15, and 20 (Figure 2).

The results showed that long-term exposure to hesperidin significantly reduced the frequency of spontaneous micronucleated cells in U87MG cultures in a passage-dependent manner. A progressive decrease in micronucleated cell frequency was observed with an increasing number of passages under hesperidin treatment. Importantly, this effect occurred in the absence of irradiation, indicating that hesperidin can reduce baseline genomic instability.

**FIGURE 3:**
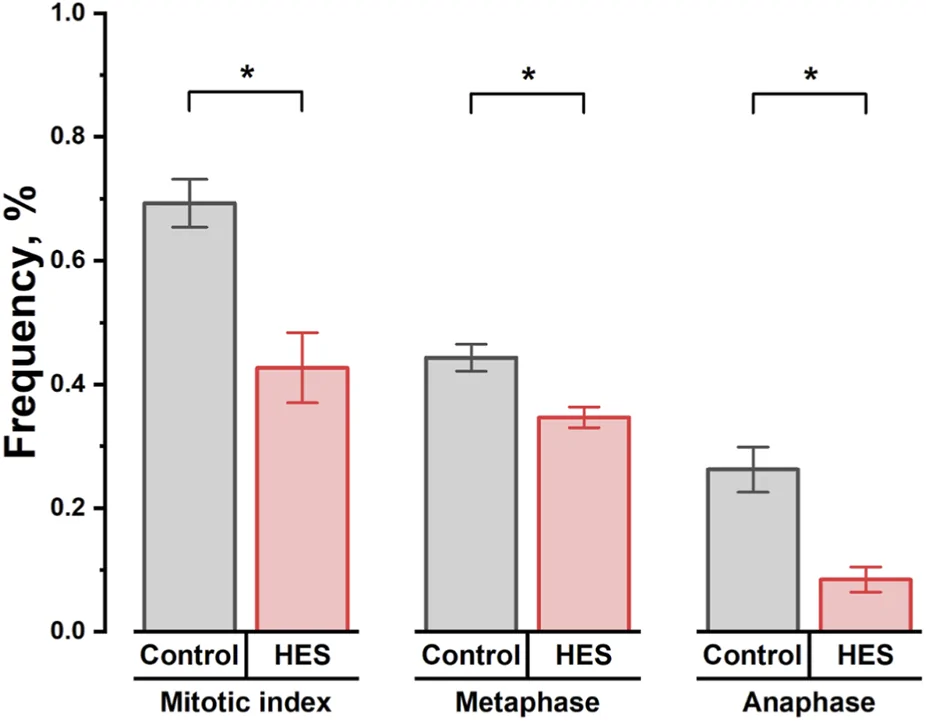
Effect of long-term hesperidin treatment on mitotic activity in U87MG glioblastoma cells. Mitotic index and the frequencies of metaphase and anaphase cells were determined following prolonged cultivation in the presence of hesperidin (HES). Hesperidin significantly reduced the overall mitotic index as well as the proportions of cells in metaphase and anaphase compared with untreated controls. Data are presented as mean ± SEM. p < 0.05 versus control.

To determine whether the observed reduction in micronucleus frequency could be associated with altered proliferative activity, the mitotic index as well as the frequencies of metaphase and anaphase cells were additionally evaluated after long-term hesperidin treatment (Figure 3). Hesperidin significantly reduced the overall mitotic index and decreased the proportion of cells undergoing metaphase and anaphase compared with untreated control cultures. No preferential accumulation of cells at a particular mitotic stage was observed, indicating an overall reduction in mitotic activity rather than arrest at a specific phase of mitosis. These observations indicate that hesperidin influences proliferative activity during long-term cultivation. Therefore, changes in cell proliferation should be considered when interpreting the reduction in spontaneous micronucleus frequency.

Taken together, these findings demonstrate that long-term hesperidin treatment was associated with reduced spontaneous micronucleus formation and decreased mitotic activity in U87MG glioblastoma cells. These observations support further investigation of hesperidin as a potential modulator of genomic instability during long-term tumor cell cultivation.

Compared with the synthetic radioprotector amifostine, hesperidin offers the advantage of a favorable safety profile, as serious side effects have been reported for the former substance (Demiral et al., 2002). In contrast, amifostine’s clinical use is limited by significant adverse effects including Stevens-Johnson syndrome and toxic epidermal necrolysis (Demiral et al., 2002). However, it is important to note that radioprotective effects of hesperidin have been demonstrated *in vitro* in the present study, and clinical translation would require extensive further evaluation. Nevertheless, hesperidin’s ability to reduce both spontaneous and radiation-induced micronucleus formation in glioblastoma cells, combined with its low toxicity profile, might indicate its potential as a promising radioprotective agent.

### 3.4 Effect of X-ray irradiation on HNRNPA2B1 expression

Dose-dependent expression of HNRNPA2B1 protein was assessed in U87MG glioblastoma cells following different doses of X-ray irradiation.

**FIGURE 4:**
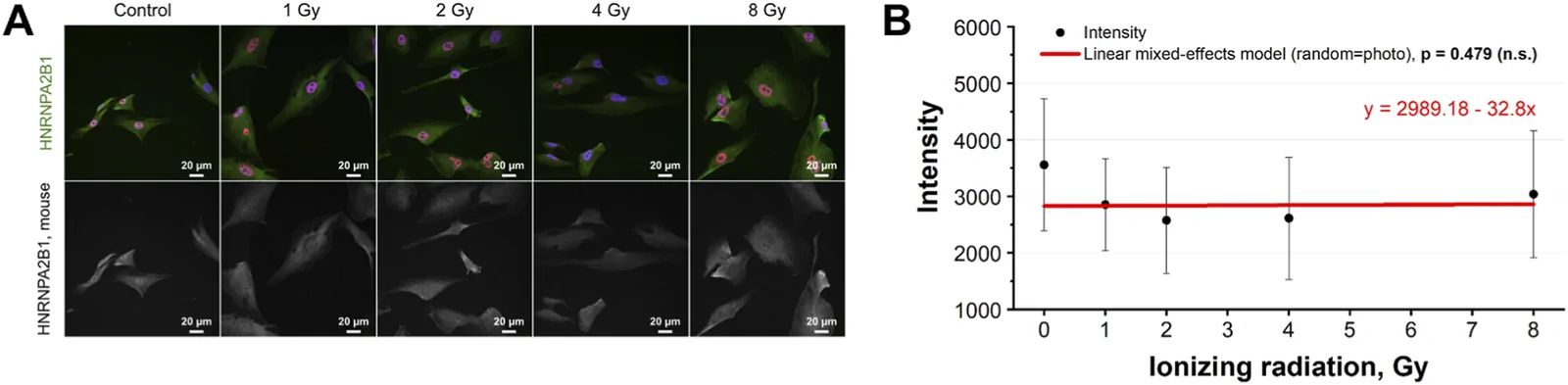
HNRNPA2B1 protein expression is not altered by ionizing radiation in U87MG glioblastoma cells. **(A)** Confocal microphotographs of U87MG cells stained for the HNRNPA2B1 protein (A2B1), demonstrating its localization in both the cytoplasm and nucleus. Cells were exposed to increasing doses of ionizing radiation (0, 1, 2, 4, and 8 Gy). Visual analysis revealed no apparent dose-dependent changes in signal intensity or subcellular distribution of the protein. **(B)** Semi-quantitative analysis of the HNRNPA2B1 fluorescent signal intensity. The plot shows no significant correlation between radiation dose and protein expression level. Linear regression analysis confirms the absence of a dose-dependent trend, indicating that total HNRNPA2B1 protein levels remain stable despite escalating radiation doses.

Immunocytochemical staining revealed no clear dose-dependent regulation of this protein. Representative microphotographs (Figure 4) illustrate its localization and signal intensity under different experimental conditions. Quantitative analysis of fluorescence intensity also showed no statistically significant differences in HNRNPA2B1 expression between samples irradiated at varying doses.

Thus, under the present experimental conditions, total HNRNPA2B1 protein expression in U87MG cells did not exhibit a dose-dependent response to acute radiation exposure. This finding indicates that the radiation-induced increase in micronuclei frequency (Figure 1A) is not accompanied by corresponding changes in HNRNPA2B1 protein levels. Whether HNRNPA2B1 function–such as its m^6^A-binding activity, interaction with partner proteins, or post-translational modifications–is affected by radiation remains to be investigated.

### 3.5 Long-term hesperidin treatment is associated with decreased HNRNPA2B1 and m^6^A levels

To investigate whether the reduction in genomic instability observed following long-term hesperidin treatment is accompanied by changes in epitranscriptomic markers, immunocytochemical staining was performed in U87MG glioblastoma cells. The selected targets were HNRNPA2B1 protein and m^6^A-modified RNA, as HNRNPA2B1 is a well-characterized reader of the m^6^A modification and plays a central role in regulating the metabolism, stability, and expression of m^6^A-modified transcripts associated with carcinogenesis and radiosensitivity.

Immunocytochemical analysis showed that prolonged hesperidin exposure was associated with reduced HNRNPA2B1 protein levels and decreased global m^6^A modification in glioblastoma cells. This reduction was evident both visually in microphotographs and quantitatively using the H-score method.

**FIGURE 5:**
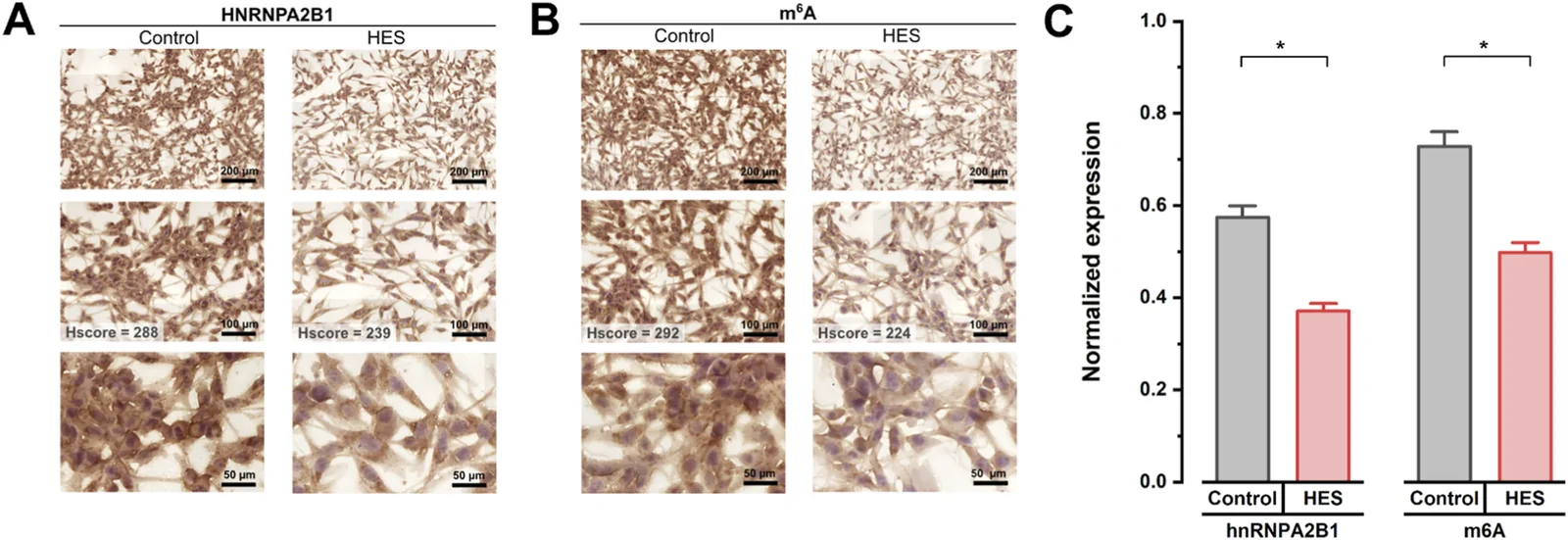
Long-term hesperidin treatment is associated with reduced HNRNPA2B1 expression and reduced global m^6^A RNA methylation levels in glioblastoma cells. **(A)** Representative immunocytochemical images of U87MG glioblastoma cells stained for the HNRNPA2B1 protein. Cells were treated with hesperidin (HES, 8 mg/mL nominal concentration) or maintained in control medium during long-term cultivation. A decrease in specific cytoplasmic/nuclear brown staining (DAB precipitate) is evident in the HES-treated group. **(B)** Representative immunocytochemical images of the same U87MG cells stained for m^6^A-modified RNA. The intensity of the brown DAB signal, representing global m^6^A levels, is reduced following hesperidin treatment. **(C)** Semi-quantitative assessment using the H-score method confirmed the visual observations. Hesperidin treatment resulted in a statistically significant decrease in the normalized signal intensity for both the HNRNPA2B1 protein and the global level of m^6^A RNA methylation (*p < 0.05). The concurrent reduction in HNRNPA2B1 protein expression and global m^6^A RNA levels indicates that long-term hesperidin treatment was accompanied by changes in these epitranscriptomic markers.

These findings demonstrate that long-term hesperidin treatment was associated with reduced HNRNPA2B1 protein expression, lower global m^6^A RNA levels, and a decreased frequency of micronucleated cells in glioblastoma cultures (Figures 5A,B). Although these observations occurred concurrently, the present study was not designed to determine whether they are causally related. Further studies employing HNRNPA2B1 gain- and loss-of-function approaches, together with transcriptome-wide m^6^A profiling, will be required to determine whether the observed molecular changes contribute to the reduction in genomic instability or represent parallel responses to hesperidin treatment.

The consistently observed reduction in the frequency of micronucleated cells in populations cultured in the presence of hesperidin may be explained by two non-exclusive mechanisms. First, hesperidin may create conditions in which clones with reduced spontaneous genomic instability acquire a selective growth advantage. Second, hesperidin may influence pathways regulating genome stability, potentially including those involving RNA methylation and HNRNPA2B1. Although the present study did not directly measure R-loops, previous work has established connections between m^6^A modification, HNRNPA2B1, and R-loop metabolism (Qiu et al., 2024; Li et al., 2023). Regardless of the precise mechanism, the observation that long-term hesperidin treatment was accompanied by reduced global m^6^A levels and decreased micronucleus frequency warrants further investigation into the molecular pathways involved.

## 4 Conclusion

This study employed complementary *in vitro* approaches to evaluate hesperidin as a potential modulator of genomic instability in tumor cells. Experiments performed in U87MG and LN229 glioblastoma cell lines demonstrated that hesperidin significantly reduced both spontaneous and radiation-induced micronucleus formation, indicating reduced genomic instability and radioprotective activity under the experimental conditions used in this study. Long-term hesperidin treatment was also accompanied by reduced HNRNPA2B1 protein expression and lower global m^6^A RNA levels. These findings further support the pleiotropic biological activity of hesperidin and extend its previously reported radiomodulatory and antitumor properties to the modulation of genomic instability and epitranscriptomic markers.

The present study was not designed to establish a causal relationship between the observed reduction in genomic instability and the accompanying changes in HNRNPA2B1 expression and global m^6^A RNA methylation. Nevertheless, our findings are consistent with previous computational studies that identified HNRNPA2B1 as a potential molecular target of hesperidin, although direct target validation remains to be performed (Lubinets et al., 2026). Future studies employing analytical-grade hesperidin, HNRNPA2B1 gain- and loss-of-function approaches, RNA immunoprecipitation sequencing, and transcriptome-wide m^6^A profiling will be required to determine whether these molecular changes contribute to the observed reduction in genomic instability or represent parallel responses to hesperidin treatment.

## Data Availability

The raw data supporting the conclusions of this article will be made available by the authors, without undue reservation.
